# Sarcomas other than Kaposi's sarcoma in immunodeficiency

**DOI:** 10.1186/1750-9378-7-S1-P23

**Published:** 2012-04-19

**Authors:** Kishor Bhatia, Meredith S Shiels, Alexandra Berg, Eric A Engels

**Affiliations:** 1 Division of Cancer Epidemiology and Genetics, National Cancer Institute, National Institutes of Health, Rockville, MD, USA

## Background

Individuals with acquired immunodeficiency are at heightened risk for multiple subtypes of cancer. Among sarcomas, increased risk in both adults and children has been identified only for Kaposi’s sarcoma (KS), and in HIV-infected children for leiomyosarcoma. In contrast, broader diversity in subtypes of lymphomas and carcinomas has been reported. Sarcomas constitute <1% of all incident cancers in the general population. Modest increases in risk of rare sarcoma categories in the immunocompromised may thus be difficult to capture. We therefore reviewed published data from case reports/series to describe sarcoma subtypes in HIV-infected individuals and solid organ transplant recipients.

## Methods

Literature review of sarcoma case reports in people with HIV and recipients of organ transplants were conducted using PubMed and citation searches. Surveillance Epidemiology and End Results (SEER) data (1974-2008) were queried to obtain counts of each type of sarcoma, and the age distribution of leiomyosarcomas.

## Results

A total of 152 cases of sarcomas (other than KS) were identified in people with HIV/AIDS (n=62) and in recipients of solid organ transplants (n=90). Leiomyosarcomas represented the bulk of all sarcomas (98/152), followed by angiosarcoma (16/152) and fibrohistiocytic tumors (15/152). Compared to the distribution of sarcomas in SEER, leiomyosarcoma was overrepresented by 4-fold (16.9% in SEER; 64.4% in immunodeficiency). Ages of HIV-related leiomyosarcoma cases suggested a bimodal distribution with an early peak among 0-9 year olds (36%) and later among 30-39 year olds (34%). More than 80% of immunodeficiency-related leiomyosarcomas were Epstein-Barr virus (EBV)-positive. Most HIV-related leiomyosarcomas (>93%) were in people with AIDS diagnoses and the majority of transplant-related leiomyosarcomas (>62%) were reported in renal transplant recipients. Leiomyomas were also reported in people with immunodeficiency (n=37), and >65% of leiomyomas and leiomyosarcomas reported in people with HIV infection occurred with CD4 counts of <50 cells/mm^3^. All 14 cases of transplant-related angiosaromas were in renal transplants. Ninety three percent (13) occurred in males. Half of transplant-related angiosarcomas occurred at the site of an arteriovenous fistula.

## Conclusions

EBV-positive smooth muscle tumors are frequently reported in people with immunosupression. Both children and adults are at risk for leiomyosarcomas. Leiomyomas and leiomyosarcomas occurred with the same propensity in people with CD4 counts below 50. Angiosarcomas occurred specifically in males in renal transplant recipients and may be related to an arteriovenous fistula.

